# A class of Bayesian methods to combine large numbers of genotyped and non-genotyped animals for whole-genome analyses

**DOI:** 10.1186/1297-9686-46-50

**Published:** 2014-09-22

**Authors:** Rohan L Fernando, Jack CM Dekkers, Dorian J Garrick

**Affiliations:** Department of Animal Science, Iowa State University, 50011 Ames, Iowa USA; Institute of Veterinary, Animal and Biomedical Sciences, Massey University, Palmerston North, New Zealand

## Abstract

**Background:**

To obtain predictions that are not biased by selection, the conditional mean of the breeding values must be computed given the data that were used for selection. When single nucleotide polymorphism (SNP) effects have a normal distribution, it can be argued that single-step best linear unbiased prediction (SS-BLUP) yields a conditional mean of the breeding values. Obtaining SS-BLUP, however, requires computing the inverse of the dense matrix G of genomic relationships, which will become infeasible as the number of genotyped animals increases. Also, computing G requires the frequencies of SNP alleles in the founders, which are not available in most situations. Furthermore, SS-BLUP is expected to perform poorly relative to variable selection models such as BayesB and BayesC as marker densities increase.

**Methods:**

A strategy is presented for Bayesian regression models (SSBR) that combines all available data from genotyped and non-genotyped animals, as in SS-BLUP, but accommodates a wider class of models. Our strategy uses imputed marker covariates for animals that are not genotyped, together with an appropriate residual genetic effect to accommodate deviations between true and imputed genotypes. Under normality, one formulation of SSBR yields results identical to SS-BLUP, but does not require computing G or its inverse and provides richer inferences. At present, Bayesian regression analyses are used with a few thousand genotyped individuals. However, when SSBR is applied to all animals in a breeding program, there will be a 100 to 200-fold increase in the number of animals and an associated 100 to 200-fold increase in computing time. Parallel computing strategies can be used to reduce computing time. In one such strategy, a 58-fold speedup was achieved using 120 cores.

**Discussion:**

In SSBR and SS-BLUP, phenotype, genotype and pedigree information are combined in a single-step. Unlike SS-BLUP, SSBR is not limited to normally distributed marker effects; it can be used when marker effects have a *t* distribution, as in BayesA, or mixture distributions, as in BayesB or BayesC *π*. Furthermore, it has the advantage that matrix inversion is not required. We have investigated parallel computing to speedup SSBR analyses so they can be used for routine applications.

**Electronic supplementary material:**

The online version of this article (doi:10.1186/1297-9686-46-50) contains supplementary material, which is available to authorized users.

## Background

Due to advances in molecular biology, high-density single nucleotide polymorphisms (SNP) data are now being incorporated with phenotypic data into genetic evaluation [1–4] in what has been called genomic prediction or genomic selection [5]. Typically, genotypes are initially available only on a few thousand individuals at many thousands to several hundred thousand SNPs. Phenotypic values or deregressed estimated breeding values (EBV) on these genotyped individuals are used to estimate the effects of the SNPs using Bayesian multiple-regression models in which the marker effects are treated as random [5]. We refer to such models as marker effect models (MEM). The estimated marker effects are then used to predict the breeding values (BV) of animals that may not yet have phenotypes but have been genotyped.

Nejati-Javaremi [6] proposed an alternative approach to incorporate genotype information into genetic evaluation, where the BV of the animals are fitted, as in a pedigree-based best linear unbiased prediction (BLUP) analysis, but with a genomic relationship matrix, computed from available genotypes, that replaces the pedigree-based additive relationship matrix. We refer to these models as breeding value models (BVM). Fernando [7] showed that the MEM and BVM provide equivalent predictions of BV and that predictions could be more easily obtained from MEM than from BVM because at the time of their study the number of markers was much smaller than the number of genotyped animals. This situation changed when high-density SNP data became available and the BVM was rediscovered for genomic selection [8, 9]. In this context, BLUP using the BVM is often referred to as G-BLUP.

When MEM are used and not all animals are genotyped, marker-based EBV are typically combined with pedigree-based BLUP EBV from the entire breeding population to improve accuracy, using various selection-index approximations [2, 10]. Legarra et al. [11–14] proposed an alternative to this approximate approach to combine information from genotyped and non-genotyped animals, where in a single step, they obtain BLUP EBV combining phenotypic, pedigree and SNP data using Henderson’s mixed model equations (MME) for a BVM with a modified version of the additive relationship matrix **H** that reflects the additional information from the SNP genotypes. Thus, their method is called single-step BLUP (SS-BLUP), and is expected to yield unbiased predictions under multivariate normality, even in populations that are undergoing selection and non-random mating. This is important, because genotypes are usually collected only on superior animals and this can lead to biased evaluations. A properly calculated BLUP evaluation has been shown to be free of this selection bias [15–21]. Because their BLUP analysis is based on a BVM, we will refer to their SS-BLUP as SSBV-BLUP.

In SSBV-BLUP, the SNP data are used to construct the matrix **G** of genomic relationships for the genotyped individuals [6, 8, 22]. Conceptually, the remaining relationship coefficients constructed from pedigree are modified to provide consistency with **G**[11]. Provided that **G**^−1^ and the inverse  of the corresponding additive relationship matrix are available, an efficient algorithm has been developed to construct **H**^−1^[13]. However, computing **G**^−1^ and  is an inefficient process, because the computing time to obtain these inverses is proportional to , where *n*_2_ is the number of individuals with genotypes. Nevertheless, when *n*_2_ is a few thousands, SSBV-BLUP provides an elegant and convenient method to estimate BV that combines the available phenotype, pedigree and SNP data. Due to wide-spread adoption of genotyping in livestock, however, *n*_2_ is becoming too large for SSBV-BLUP to remain computationally feasible much longer [23, 24]. One strategy to overcome this problem is to approximate **G** such that the inverse could be computed efficiently [24]. Another strategy is to obtain solutions to the MME without explicitly inverting **G**[23]. Because **G** is very dense and it grows in size as more individuals are genotyped, these strategies are not very promising.

As discussed by Stranden and Garrick [9], when the number of genotyped individuals exceeds the number of marker covariates, use of MEM, which do not require computing **G** or its inverse, will lead to more efficient calculations. At present, however, most analyses that use MEM are based on Markov chain Monte-Carlo (MCMC) techniques that are computationally demanding. In addition, analyses using MEM have not been able to accommodate animals without genotypes.

In this paper, we extend MEM to accommodate animals without genotypes and propose alternative MCMC approaches to address computing requirements. The methodology presented here will extend the attractive features of SSBV-BLUP to Bayesian multiple-regression analyses that draw inferences from posterior distributions using MCMC techniques. Our extended MEM will enable BLUP evaluations without having to compute **G** or its inverse, while combining information from genotyped and non-genotyped animals.

## Methods

We begin this section with a short introduction to the most widely used MEM and equivalent BVM. Then, we briefly review the theory that underlies SSBV-BLUP and show that the BVM used in SSBV-BLUP is equivalent to an MEM that can be used for single-step Bayesian regression (SSBR). Finally, strategies will be presented to implement a Gibbs sampler for drawing inferences from the posterior distributions of the breeding values.

### Marker effect models

The groundbreaking paper of Meuwissen et al. [5] proposed three multiple-regression, MEM for genomic selection. These models can be described by the following general model:
1

where **y** is the vector of trait phenotypes, **X** is a known incidence matrix that relates the vector of non-genetic, “fixed” effects to **y**, **T**=**M**−*E*(**M**), **M** is a matrix of marker covariates, ***α*** is the vector of random, partial-regression coefficients of the marker covariates, and **e** is a vector of residuals. The expected value of the marker covariates can be written as *E*(**M**)=**1k**^′^, where the row vector **k**^′^ is the vector of expected values of marker covariates for a random animal in the absence of selection.

Meuwissen et al. [5] actually used haplotype covariates in their model, but now most analyses are based on SNP marker covariates. Their model assumed that the markers completely capture the first and second moments of the BV. When this is not true, a polygenic residual BV can be added to equation (1). Strandén and Christensen showed that different allele coding methods lead to the same inference of marker effects when the general mean is included in the model and all genotypes are observed [25]. However, centered allele coding, as in matrix **T**, had a numerical advantage when MCMC methods were used [25].

In the Bayesian implementation of the MEM, the fixed effects are usually given a flat prior. The *α*_*j*_ are a priori assumed independently distributed as:
2

where  are a priori assumed identically and independently distributed (iid) scaled inverted chi-square variables with scale  and degrees of freedom *ν*_*α*_. The residuals are typically assumed iid normal with null mean and variance , with a scaled inverted chi-square prior for , with scale  and degrees of freedom *ν*_*e*_.

In the first model considered by Meuwissen et al. [5], the *α*_*j*_ were iid normal variables with null mean and a common “known” variance, which is equivalent to our general model with *π*=0, , and  set to the common known variance. This model was called “BLUP” in their paper [5]. The second model that they considered (BayesA) is equivalent to our general model with *π*=0, and  and *ν*_*α*_ set to “known” values. Their third model (BayesB) is identical to BayesA, except with *π*=0.95 or some other “known” value.

Kizilkaya et al. [26, 27] modified the “BLUP” model to have a value of *π*>0 and an unknown common variance with a scaled inverted chi-square prior and referred to this model as BayesC. Furthermore, Habier et al. [27] extended the BayesC model in which the value of *π* is unknown with a uniform prior and referred to this as BayesC *π*. In Bayesian Lasso, the marker effects are assigned a double exponential prior. This can be achieved by setting *π* in equation (2) to 0 and using an exponential prior distribution for [28].

Inferences on the breeding values and other unknown parameters in the model are made from their marginal posterior distributions, using MCMC methods [5, 27, 28]. Let **t***c*′ be the row vector of marker covariates for some selection candidate. Then, the conditional mean for its genomic EBV is , where  is the posterior mean of ***α***, which can be computed from the MCMC samples.

### Breeding value models

Two mixed linear models are said to be linearly equivalent if the vector **y** of observations has the same first and second moments in both models [29]. In that sense, a model that is equivalent to (1) can be written as:
3

where **g**=**T*α***, and **T**=**M**−*E*(**M**) is the matrix of centered marker covariates. One of the advantages of using centered marker covariates is that the vector **g** of breeding values will have null means even if ***α*** does not have null means. The covariance matrix of the breeding values is:


Then, in both models (1) and (3), the mean of **y** is **X*β*** and the covariance matrix is:


Thus, these two models are linearly equivalent and the parameters of one model can always be written as linear functions of the parameters of the other model. “Consequently, linear and quadratic estimates under one model can be converted by these same linear functions to estimates of an equivalent model” [29].

When the number of markers is large relative to the size of **g**, BLUP of **g** can be obtained efficiently [8, 9] by solving the MME that correspond to model (3). When *π*=0 and  in (1), the covariance matrix of marker effects is:


and the covariance matrix of the genomic BV conditional on **M** can be written as:
4

Furthermore, under some assumptions, the variance  of marker effects can be related to the variance  of BV as:
5

where *p*_*j*_ is the frequency of SNP *j*[22, 30, 31]. Then, equation (4) can be written as:
6

Suppose genotypes were not available and the analysis is conditional only on pedigree, which we denote as **P**. Then, the conditional mean of **g** given **P** is null and the conditional covariance matrix is:


where **A** is the additive relationship matrix, and the variance is computed over the conditional distribution of **T**. Using this variance for **g** in setting up the MME for equation (3) results in the usual non-genomic MME for the BVM.

### Theory underlying SSBV-BLUP

Legarra et al. [11] proposed an ingenious strategy to combine information from genotyped and non-genotyped animals in a single BLUP analysis based on a BVM, which we refer to as SSBV-BLUP. Suppose **g** is partitioned as:


where *g*_1_ are BV of the animals with missing genotypes **T**_1_ and **g**_2_ are BV of those with observed genotypes **T**_2_. Following Legarra et al. [11], the vector **g**_1_ can be written as:
7

where **A**_*ij*_ are partitions of **A** that correspond to **g**_1_ and **g**_2_. The first term in equation (7) is the best linear predictor (BLP) of **g**_1_ given **g**_2_, and the second is a residual genetic effect to accommodate deviations between the true breeding value, **g**_1_, and its prediction from **g**_2_, , which we refer to as ***ε***, the “imputation residual”.

Consider first the conditional distribution of **g**_1_ given **P**. Then, as expected, the variance of **g**_1_ is:
89

where the first term of equation (8) is the variance of the  (i.e. predicted from its relatives in **g**_2_) and the second term is the variance of ***ε***. Similarly, .

In this situation, where the covariance structure of **g**_1_ and **g**_2_ is determined entirely by the pedigree, it is easy to see that ***ε*** in equation (7) is uncorrelated to **g**_2_, and therefore if **g**_1_ and **g**_2_ are multivariate normal, ***ε*** and **g**_2_ are independent. Multivariate normality of **g**_1_, **g**_2_ and consequently of ***ε*** will be a good approximation if the effective number of loci that contribute to the BV is large. This will be the case even when the individual marker effects do not have a normal distribution, as in BayesA and BayesB.

Consider now the conditional distribution of **g**_1_ given **T**_2_. Note that, given the observed genotypes **T**_2_, the distribution of **g**_2_ changes to a multivariate normal with mean **0** and covariance matrix . Now, to obtain the result in [11] for the conditional distribution of **g**_1_ given **T**_2_, we have to assume that the change in the distribution of **g**_1_ occurs entirely as a result of the change in the distribution of **g**_2_. In other words, we only use the information in **T**_2_ that flows to **g**_1_ through **g**_2_ (see Discussion). Then, it can be reasoned that:
10

where now the vector  has covariance matrix given by the first term of equation (10), and because ***ε*** is independent of **g**_2_, the second term of equation (10) remains identical to that of equation (8). Similarly, the covariance between **g**_1_ and **g**_2_ conditional on **T**_2_ is:


Furthermore, assuming equation (5), the above results can be combined to show that conditional on **T**_2_, **g** has a multivariate normal distribution with null mean and covariance matrix:
11

where . An alternative derivation of this matrix was given by Christensen and Lund [14]. The inverse of this matrix is needed to set up the MME, and can be computed as


which is the basis for SSBV-BLUP [11, 13, 14]. Note that this requires that both **G**^−1^ and  are computed beforehand, which is not computationally feasible because these matrices are dense and large when the number *n*_2_ of genotyped animals is large. Furthermore, **T**_2_**T**2′ is not full rank when *n*_2_ exceeds the number of markers. Thus to obtain a full rank **G**, ad-hoc adjustments are often made, such as adding small values to the diagonals or regressing **G** towards **A**, which is justified when the markers do not capture all the genetic variability. However, due to the increased adoption of SNP genotyping in livestock, **G**^−1^ and  are becoming too large for SSBV-BLUP to remain computationally feasible [23, 24]. A second problem in SSBV-BLUP is related to the scaling that is done using the SNP frequencies. As mentioned earlier, when all data that were used for selection are available for computing the conditional mean, it can be computed as if selection had not taken place [18, 20, 21]. If selection has taken place, this requires using SNP frequencies from the founders, because these frequencies are not changed by selection. However, in most situations SNP genotypes are not available in the founders and frequencies observed in the genotyped animals are used, which can lead to biased evaluations, particularly in a multi-breed context.

### Single-step Bayesian regression

Assuming that BV are captured completely by marker genotypes, the mixed linear model for the phenotypic values can be expressed in terms of a BVM (12) or an MEM (13) as
1213

where we have introduced the incidence matrix **Z** to accommodate animals with repeated records or animals without records. As in SSBV-BLUP, suppose **T**_1_ is not observed. Then it is not possible to use equation (13) as the basis for the MEM. Note that **T**_1_***α*** is equal to **g**_1_. So, using equation (7) for **g**_1_ and writing **g**_2_=**T**_2_***α***, the model for the phenotypic values becomes:
1415

To construct the matrix **T**_2_ of centered marker covariates, we need to know the value of *E*(**M**_2_)=**1k**^′^, where the row vector **k**^′^ is the vector of expected values of marker covariates for a random animal in the absence of selection. However, marker covariates are often available only for animals that have been subject to selection. So, we propose to write **g**_2_ as:
161718

where *μ*_*g*_=**k**^′^***α*** is assigned a flat prior. The reason *μ*_*g*_ can be considered an independent parameter is that **k**^′^ is a vector of unknown parameters. Substituting equation (18) for **g**_2_=**T**_2_***α*** in equation (15) gives:
1920

where , , , **J**_2_=−**1**, ,


The matrix  of imputed marker covariates can be obtained efficiently, using partitioned inverse results, by solving the easily formed very sparse system [see Additional file 1]:

21

where **A**^*ij*^ are partitions of **A**^−1^ that correspond to partitioning **g** into **g**_1_ and **g**_2_. Similarly, the vector  can be obtained efficiently by solving the system:
22

The differences between this MEM (20) and the model that is currently used for Bayesian regression (BR) are: (i) estimation of an extra fixed effect: *μ*_*g*_=**k**^′^***α***, (ii) some of the marker covariates in (20) are imputed, and (iii) a residual term ***ε*** has been introduced to account for deviations of the imputed marker covariates from their unobserved, actual values.

When genotypes are not missing, the vector **J**_1_ is null, and the covariate for *μ*_*g*_ only contains **J**_2_, which is in the column space of **X**_2_ when the model explicitly or implicitly contains the general mean. In this case, it has been shown that inference on differences between breeding values is not affected by the choice of vector **k**^′^ used to center the marker covariates [25]. This includes using **k**^′^=**0**^′^, which corresponds to not centering, and therefore, *μ*_*g*_ does not have to be included in the model. However, when genotypes are missing  may not be in the column space of **X**_1_, and thus *μ*_*g*_ must be included in the model if **k**^′^ is not known and the marker covariates are not centered. A thorough investigation of this approach of including *μ*_*g*_ as a fixed effect in the model in place of centering the marker covariates is beyond the scope of this paper, but a small simulation is included here to compare the accuracy of prediction when marker covariates are centered with those when marker covariates are not centered but *μ*_*g*_ is included in the model.

Regardless of the prior used for ***α***, the distribution of the vector ***ε*** of imputation residuals will be approximated by a multivariate normal vector with null mean and covariance matrix  (see equation 10), where  is assigned a scaled inverse chi-square distribution with scale parameter  and degrees of freedom *ν*_*g*_. Imputing the marker covariates needs to be done only once, and it can be done efficiently in parallel. Imputation of unobserved marker covariates will not significantly increase the overall computing time, and the storage costs will not be greater than for centered observed genotypes.

The MME that correspond to equation (20) for BayesC with *π*=0 are
23

The submatrix of these MME that correspond to ***ε*** are identical to those for **g**_1_ from a pedigree-based analysis and are very sparse. Thus, as explained in the next section, conditional on ***β***^∗^ and ***α***, ***ε*** can be sampled efficiently by using either a blocking-Gibbs sampler [32, 33] or a single-site Gibbs sampler, as used in pedigree-based analyses [33]. Note that these MME, which do not have **G** or its inverse, may be used to overcome the computational problems with SSBV-BLUP. The predicted BV can be written as:
24

The MME given by equation (23) have the advantage that they will not grow in size as more animals are genotyped, in contrast to the MME corresponding to equation (14) that are given by Aguilar et al. [13]. Results from solving equation (23) will not be identical to those from the MME corresponding to equation (14) because in equation (23), **k**^′^ is treated as an unknown, and *μ*_*g*_=**k**^′^***α*** is estimated from the genotypic and phenotypic data. However, the MME corresponding to:
25

where , and **a**_2_=**M**_2_***α*** will give predictions for breeding values computed as:
26

identical to those from equation (24).

#### Numerical example

Consider the pedigree in Table 1, and assume genotypes are available only on individuals 1, 2, and 4. Genotypes **M**_2_ at 10 markers are in Table 2. Following Legarra et al. [11], the relationship matrix is rearranged such that **A**_11_ are relationships among individuals 3, 5, and 6, which do not have genotypes, and **A**_22_ are relationships among 1, 2, and 4, which have the genotypes in Table 2. The inverse of the rearranged relationship matrix is given in Table 3. The imputed genotypes, , and the marker covariates, **J**_1_, for *μ*_*g*_ of the non-genotyped animals, could be obtained efficiently by solving the sparse systems (21) and (22), respectively (Table 4). To set up the MME, we will assume that , , and that *μ*, and *μ*_*g*_ are the only fixed effects. Then, the MME (23) and solutions corresponding to the MEM (20) are in Table 5. For comparison, the MME and solutions for the single-step BV model are given in Table 6. The solutions for *μ* and *μ*_*g*_ are identical for the two sets of MME. The BLUP of **g** obtained as equation (24), using the solutions to equation (23), are identical to those obtained from equation (26), and are in Table 1.

**Table 1:** **Pedigree used in the numerical example**

Individual	Sire	Dam	Phenotypes	SS-BLUP-BV
1	0	0	–	1.61
2	0	0	1.25	1.59
3	0	0	-0.34	0.00
4	1	2	1.30	1.62
5	1	2	1.27	1.61
6	1	3	0.46	0.80

Genotypes are available for individuals 1, 2 and 4. Phenotypes are available for all individuals except individual 1, the sire. Single-step, BLUP predictions of breeding values (SS-BLUP-BV) are in the last column.

**Table 2:** **Observed genotypes at ten markers for individuals in the example in Table** 1

Individual	m1	m2	m3	m4	m5	m6	m7	m8	m9	m10
1	1	2	1	1	0	0	1	2	1	0
2	2	1	1	1	2	0	1	1	1	1
4	1	1	0	1	1	0	2	1	2	1

**Table 3:** **Inverse of rearranged relationship matrix for individuals in the example in Table** 1

	3	5	6	1	2	4
3	1.50	0.00	-1.00	0.50	0.00	0.00
5	0.00	2.00	0.00	-1.00	-1.00	0.00
6	-1.00	0.00	2.00	-1.00	0.00	0.00
1	0.50	-1.00	-1.00	2.50	1.00	-1.00
2	0.00	-1.00	0.00	1.00	2.00	-1.00
4	0.00	0.00	0.00	-1.00	-1.00	2.00

Row and column labels are the individual identifiers.

**Table 4:** **Imputed genotypes at ten markers and covariates for** ***μ***_***g***_ **for individuals in the example in Table** 1

Individual	m1	m2	m3	m4	m5	m6	m7	m8	m9	m10	***μ***_***g***_
3	0.0	0.0	0.0	0.0	0.0	0.0	0.0	0.0	0.0	0.0	0.00
5	1.5	1.5	1.0	1.0	1.0	0.0	1.0	1.5	1.0	0.5	-1.00
6	0.5	1.0	0.5	0.5	0.0	0.0	0.5	1.0	0.5	0.0	-0.50

**Table 5:** **Mixed model equations for marker effects model with observed and imputed marker covariates for the example in Table** 1

	***μ***	***μ***_***g***_	***m***_1_	***m***_2_	***m***_3_	***m***_4_	***m***_5_	***m***_6_	***m***_7_	***m***_8_	***m***_9_	***m***_10_	***ε***_3_	***ε***_5_	***ε***_6_
*μ*	5.00	-3.50	5.00	4.50	2.50	3.50	4.00	0.00	4.50	4.50	4.50	2.50	1.00	1.00	1.00
*μ*_*g*_	-3.50	3.25	-4.75	-4.00	-2.25	-3.25	-4.00	0.00	-4.25	-4.00	-4.25	-2.50	0.00	-1.00	-0.50
*m*_1_	5.00	-4.75	8.61	5.75	3.75	4.75	6.50	0.00	5.75	5.75	5.75	3.75	0.00	1.50	0.50
*m*_2_	4.50	-4.00	5.75	6.36	3.00	4.00	4.50	0.00	5.00	5.25	5.00	2.75	0.00	1.50	1.00
*m*_3_	2.50	-2.25	3.75	3.00	3.36	2.25	3.00	0.00	2.25	3.00	2.25	1.50	0.00	1.00	0.50
*m*_4_	3.50	-3.25	4.75	4.00	2.25	4.36	4.00	0.00	4.25	4.00	4.25	2.50	0.00	1.00	0.50
*m*_5_	4.00	-4.00	6.50	4.50	3.00	4.00	7.11	0.00	5.00	4.50	5.00	3.50	0.00	1.00	0.00
*m*_6_	0.00	0.00	0.00	0.00	0.00	0.00	0.00	1.11	0.00	0.00	0.00	0.00	0.00	0.00	0.00
*m*_7_	4.50	-4.25	5.75	5.00	2.25	4.25	5.00	0.00	7.36	5.00	6.25	3.50	0.00	1.00	0.50
*m*_8_	4.50	-4.00	5.75	5.25	3.00	4.00	4.50	0.00	5.00	6.36	5.00	2.75	0.00	1.50	1.00
*m*_9_	4.50	-4.25	5.75	5.00	2.25	4.25	5.00	0.00	6.25	5.00	7.36	3.50	0.00	1.00	0.50
*m*_10_	2.50	-2.50	3.75	2.75	1.50	2.50	3.50	0.00	3.50	2.75	3.50	3.36	0.00	0.50	0.00
*ε*_3_	1.00	0.00	0.00	0.00	0.00	0.00	0.00	0.00	0.00	0.00	0.00	0.00	1.17	0.00	-0.11
*ε*_5_	1.00	-1.00	1.50	1.50	1.00	1.00	1.00	0.00	1.00	1.50	1.00	0.50	0.00	1.22	0.00
*ε*_6_	1.00	-0.50	0.50	1.00	0.50	0.50	0.00	0.00	0.50	1.00	0.50	0.00	-0.11	0.00	1.22
rhs	3.94	-4.04	5.93	4.91	2.75	4.04	5.06	0.00	5.34	4.91	5.34	3.18	-0.34	1.27	0.46
sol	-0.34	-1.61	-0.01	-0.00	-0.01	-0.00	-0.01	0.00	0.01	-0.00	0.01	0.00	-0.00	0.00	-0.01

The last two rows give the right-hand-side and the solutions of the equations.

**Table 6:** **Mixed model equations for single-step BV model for the example in Table** 1

	***μ***	***μ***_***g***_	***a***_3_	***a***_5_	***a***_6_	***a***_1_	***a***_2_	***a***_4_
*μ*	5.00	-3.50	1.00	1.00	1.00	0.00	1.00	1.00
*μ*_*g*_	-3.50	3.25	0.00	-1.00	-0.50	0.00	-1.00	-1.00
*a*_3_	1.00	0.00	1.17	0.00	-0.11	0.06	0.00	0.00
*a*_5_	1.00	-1.00	0.00	1.22	0.00	-0.11	-0.11	0.00
*a*_6_	1.00	-0.50	-0.11	0.00	1.22	-0.11	0.00	0.00
*a*_1_	0.00	0.00	0.06	-0.11	-0.11	0.32	-0.01	-0.09
*a*_2_	1.00	-1.00	0.00	-0.11	0.00	-0.01	1.31	-0.17
*a*_4_	1.00	-1.00	0.00	0.00	0.00	-0.09	-0.17	1.29
rhs	3.94	-4.04	-0.34	1.27	0.46	0.00	1.25	1.30
sol	-0.34	-1.61	-0.00	-0.01	-0.01	-0.00	-0.02	0.01

The last two rows give the right-hand-side and the solutions of the equations.

#### Simulation to compare accuracy of prediction with and without centering of covariates

Accuracy of prediction was computed for SS-BLUP with and without centering of marker covariates. As demonstrated by the preceding numerical example, the MEM given by (20) or the BVM given by (25) can be used to get identical SS-BLUP predictions. When marker covariates were not centered, accuracy was computed with and without fitting *μ*_*g*_ in the model. The simulated data consisted of 20 paternal halfsib families, each with 20 offspring. Thus, the pedigree consisted of 20 unrelated sires, 400 unrelated dams and 400 offspring. Only genotypes from the 400 offspring were used in the analysis. Phenotypes from all 400 offspring and from 210 dams were used. Results are given for four scenarios of the simulation. In all four scenarios, the trait was determined by 50 QTL and had a heritability of 0.5. All QTL effects were sampled from a normal distribution with either mean *μ*_*α*_=0 or mean *μ*_*α*_=0.2. The analysis was based on either 100 or 10 000 marker genotypes, including the QTL. Correlations between the predicted and true breeding values for the three analyses and the four scenarios are in Table 7, which contains correlations for the non-genotyped animals, and Table 8, which contains the correlations for the genotyped animals. All three models had almost identical accuracies except when the number of observations exceeded the number of markers and the marker effects had a non-null mean. In this case, the model with centered marker covariates had a higher accuracy. The same accuracy could be achieved by including an extra covariate to model the mean of the breeding values. The results in this simulation indicate that this is necessary only when the number of observations exceeds the number of markers. When the number of markers greatly exceeds the number of observations, this extra covariate may become unnecessary even when the marker effects have a non-null mean.

**Table 7:** **Correlation between predicted and true breeding values of non-genotyped animals for three models**

Markers	***μ***_***α***_	Correlations		
		CC	CN ***μ***_***g***_	CN
100	0.0	0.67	0.67	0.66
100	0.2	0.67	0.67	0.59
10,000	0.0	0.60	0.60	0.60
10,000	0.2	0.58	0.58	0.58

Models with marker covariates centered (CC), marker covariates not centered with *μ*_*g*_ in the model (CN *μ*_*g*_), and marker covariates not centered without *μ*_*g*_ (CN) in the model. The QTL effects were sampled from a normal distribution with either mean *μ*_*α*_= 0 or mean *μ*_*α*_= 0.2. The analyses were based on either 100 or 10 000 marker genotypes, including the QTL.

**Table 8:** **Correlation between predicted and true breeding values of genotyped animals for three models**

Markers	***μ***_***α***_	Correlations		
		CC	CN ***μ***_***g***_	CN
100	0.0	0.93	0.93	0.91
100	0.2	0.92	0.92	0.78
10,000	0.0	0.71	0.71	0.71
10,000	0.2	0.76	0.76	0.76

Models with marker covariates centered (CC), marker covariates not centered with *μ*_*g*_ in the model (CN *μ*_*g*_), and marker covariates not centered without *μ*_*g*_ (CN) in the model. The QTL effects were sampled from a normal distribution with either mean *μ*_*α*_= 0 or mean *μ*_*α*_= 0.2. The analyses were based on either 100 or 10 000 marker genotypes, including the QTL.

### Strategies to implement a Gibbs sampler

In most implementations of BR, a Gibbs sampler is used to draw inferences from the posterior distribution of the unknowns [5, 27, 33, 34]. This involves sampling from full-conditional posterior distributions. Sampling of fixed effects, ***β***, is almost identical to sampling the marker effects, ***α***[34]. Thus, we will describe the strategy for sampling ***α*** directly.

#### Sampling marker effects and variances

The most time-consuming task in a BR analysis is sampling ***α*** from its full-conditional distribution. Detailed derivation of these full-conditionals and illustrative R scripts are in Fernando and Garrick [34] for BR models with complete genotype data. As shown in equation (10) of [34], the first step in sampling *α*_*i*_ is adjusting **y** for all other effects in the model. In the case of equation (20), , the vector of adjusted phenotypic values is:
27

computed with the current values of ***β***^∗^, ***α*** and ***ε***. This vector is used to compute the right-hand-side for *α*_*i*_, , which is needed to sample *α*_*i*_ in BayesA, BayesB and BayesC [5, 34]. In BayesB and BayesC, *α*_*i*_ is either null or, conditional on the effect variance, normally distributed. Thus before *α*_*i*_ is sampled, a Bernoulli variable *δ*_*i*_ is sampled that indicates whether *α*_*i*_ is null or is normally distributed. Sampling *δ*_*i*_ requires computing the full-conditional probability that *δ*_*i*_=1, which also requires *rhs*_*i*_[34].

Calculation of *rhs*_*i*_ can be done more efficiently [35] by initially computing:
28

which is the vector **y** corrected for all effects in the model, using their current values. Then, before sampling *α*_*i*_, the right-hand-side for *α*_*i*_, , is obtained efficiently as:
29

and after sampling *α*_*i*_,  is updated as:
30

where  and  are the values of *α*_*i*_ before and after sampling. Then, sampling proceeds to the next locus. Thus for each marker covariate, *rhs*_*i*_ is always computed, whereas in BayesB and BayesC, the vector  only needs to be updated when . In computing *rhs*_*i*_, the first term is the dot product of two vectors of length *n*, the number of records. In the second term, the scalar **w***i*′**w**_*i*_ does not change from one round of sampling to the next, and so it is computed only once for each marker covariate. To speed-up computations, the dot product can be done in parallel, using the message passing interface (MPI) [36]. Suppose *m* processors are used for computing. Then, the first *q* elements of each covariate will be stored in memory of the first processor and the second set of *q* elements in the second processor and so on, where *q* is the whole part of , and the last processor gets the remaining elements. Similarly, blocks of  will also be stored with the *m* processors. Then, to compute , each of the *m*−1 processors will do a dot product of length *q* and the last processor one of length ≤*q*. Only the scalar result of the dot product from each processor needs to be communicated for the sampling and this will be relatively fast. After sampling *α*_*i*_, updating  will also be done in parallel. Before updating , the scalar  must be available to all *m* processors. Then, each processor will update its own block of  and will be ready for sampling the effect of the next marker covariate. The remaining calculations related to sampling marker effects do not depend on the number of records and will take only a negligible amount of time. Once the marker effects are sampled, sampling the locus-specific variances of marker effects in BayesA and BayesB, or the common variance in BayesC does not depend on the number of observations [5, 34].

#### Parallel computations

To investigate the speedup from parallel computations, the Lonestar Linux cluster of the Texas Advanced Computing Center was used. According to the documentation at (http://www.tacc.utexas.edu/user-services/user-guides/lonestar-user-guide#overview) a regular compute node on this cluster contains two Xeon Intel Hexa-Core 64-bit Westmere processors (12 cores in all) on a single board, as an SMP unit. The core frequency is 3.33 GHz and supports four floating-point operations per clock period, with a peak performance of 13.3 GFLOPS/core or 160 GFLOPS/node. Each node contains 24 GB of memory (2 GB/core). The memory subsystem has three channels from each processor’s memory controller to 3 DDR3 ECC DIMMS, running at 1333 MHz. The processor interconnect, QPI, runs at 6.4 GT/s.

Initially, a data set with 1 million individuals and 5000 markers on each individual was used. Obtaining 100 samples of ***α*** and  for BayesC with *π*=0 took 1167 seconds on a single core. By extrapolation, ignoring memory limitations, 100 samples for 50 000 markers would take about 11 670 seconds on a single core. Next, MPI [36] was used for parallel computation on 120 cores across 10 nodes. Then, obtaining 100 samples for 1 million individuals with 50 000 markers took 202 seconds. Thus, the speedup on 120 cores was about 58 times, and obtaining 40 000 samples would take about 22 hours.

#### Sampling imputation residuals and genetic variance

Using results from [33], it can be shown that conditional on the sampled value of all other variables and the data, the conditional distribution of ***ε*** is multivariate normal with mean  that is given by the solution to the following system:
31

and covariance matrix: . The right-hand-side of equation (31) can be obtained efficiently as , where  is the current value of the sub-vector from equation (28) that corresponds to **y**_1_. A sample from this distribution can be obtained by using the blocking-Gibbs sampler described by Garcia-Cortes and Sorensen [32, 33]. This requires solving equation (31), which is very sparse, and can be done iteratively. Alternatively, a single-site Gibbs sampler can be used [33]. It can be shown that the full-conditional posterior for  is a scaled inverse chi-square distribution with scale parameter:


and degrees of freedom , where *n*_*ε*_ is the number of elements in *ε*[33]. The matrix **A**^11^ is very sparse, and thus computing ***ε***^′^**A**^11^***ε*** is fast.

### An alternative sampler

Here, we consider the situation where ***ε*** is a “nuisance parameter” and interest is only on inference about marker effects: ***α***. The starting point for this sampler is the MME given by equation (23). When *π*=0 in the prior of marker effects of equation (2), conditional on the variance components in the model, the posterior for the location parameters is a normal distribution with mean given by the solution to these MME and covariance matrix given by the the inverse of the left-hand-side of the MME times [33]. Eliminating ***ε*** from (23) results in the following MME:
32

where . The solution to these MME for  and  are identical to those from equation (23). Furthermore, the inverse elements of the left-hand-side of these MME are identical to the corresponding inverse elements of equation (23). Thus, equation (32) can be used to draw samples from the posterior of ***β*** and ***α***.

In BayesA and BayesC with *π*=0, the blocking-Gibbs sampler of [32] can be used to sample all effects jointly. In BayesB, BayesC and BayesC *π*, the single-site sampler is more convenient. When the number of observations is larger than the number of effects in the model, equation (13.12) of [33] can be used to compute *rhs*_*i*_ efficiently, which is required to sample the *i*^*th*^ location effect from its full-conditional posterior distribution [5, 34] as:
33

where *r*_*i*_ is the *i*^*th*^ element from the right-hand side of equation (32), **C***i*′ is the *i*^*th*^ row from the left-hand side of equation (32), and ***θ*** is the vector of sampled values of the fixed effects and marker effects. Once equation (32) is set up, the time for computing *rhs*_*i*_ as equation (33) does not depend on the number of observations. Thus, computing time for sampling ***β*** and ***α*** by either the blocking-Gibbs sampler or by the single-site sampler, using equation (33) to compute *rhs*_*i*_, does not depend the number on observations.

Furthermore, when *π* is close to 1, the sampled value for most marker effects is null. Then, dramatic reductions in computing time can be achieved as described in the following. Sampling is started with ***θ=0***, and so initially, the vector **rhs**=**r**. As sampling proceeds and any non-null effect *θ*_*i*_ is sampled, **rhs** is updated as:
34

and


where **C**_*i*_ is the *i*^*th*^ column of **C**. Then, before sampling *θ*_*i*_, *rhs*_*i*_ would be equal to equation (33). In sampling marker effects, updating **rhs** using equation (34) is the only non-scalar computation, and when *π* is close to 1, the number of such updates can be very small. In such situations, 40k samples of BayesB can be obtained on a single core in about half an hour, regardless of the number of observations.

The most intensive computation in setting up equation (32) is that of **W**1′**PW**_1_ and **W**2′**W**_2_. First, we consider computing the matrix of crossproducts: **W**2′**W**_2_. For an arbitrary matrix **S** of *n* rows and *k* columns, the crossproduct **S**^′^**S** can be written as:
35

where  In equation (35), the crossproducts are independent and can be done in parallel.

Next, we consider computing **W**1′**PW**_1_. This can be undertaken in two steps. In step 1, the columns of **B**=**PW**_1_ are computed in parallel. Column *i* of this product can be written as:


where **W**_1*i*_ is the *i*^*th*^ column of **W**_1_. The second term in **b**_*i*_ can be computed efficiently as **Z**_1_**q**_*i*_, where **q**_*i*_ is the solution to the sparse system:


The Cholesky decomposition of this system is also sufficiently sparse for exact computation and has to be done only once. Once **B** is computed, in the second step, the product **W**1′**PW**_1_=**W**1′**B** can be computed in parallel, similar to equation (35), as the sum of independent matrix products.

#### Parallel computations

The Lonestar Linux cluster was used to examine the possible speedup that could be achieved by parallel computing of **S**^′^**S**. Initially, a single core on this cluster was used to compute **S**^′^**S** with the number of rows in **S**, *n*, equal to 1 million and the number of columns, *k* equal to 5000. This computation took 11 669 seconds. If memory was not limiting, computation with *k* = 50 000 would take 100 times longer because now **S**^′^**S** would be 100 times larger than with *k*=5000. Actual calculations with *k* = 50 000 were undertaken with 200 nodes. In each node, **S***i*′**S**_*i*_ was computed with **S**_*i*_ being a slice containing a subset of the 50 000 rows of the **S** matrix. MPI [36] was used to compute **S**^′^**S** as the sum of these matrices, as in equation (35). The Eigen C++ template library [37], which can exploit the multiple cores within a node, was used to compute **S***i*′**S**_*i*_ within each node. Although each node of the Lonestar cluster has 12 cores, using 8 cores within each node gave the best result: 912 seconds to compute **S**^′^**S**. This was a speedup of about 1,279 times.

## Discussion

Genomic prediction is based either on marker effects models (MEM), where the effects of marker covariates are explicitly included in the model as random effects, or on breeding value models (BVM), where the markers are used to compute the covariance matrix of the breeding values. Although BLUP using these two types of models can be identical [9], computing using the BVM is more efficient when the number of marker covariates is much larger than the number of individuals. Furthermore, the BVM has also been used to combine information from genotyped and non-genotyped individuals to obtain BLUP in a single step (SSBV-BLUP) [11, 13, 14].

SSBV-BLUP is based on the conditional covariance matrix, **H**, of **g** given both pedigree and observed marker genotypes. When this covariance matrix is written in terms of a single variance component, as in equation (11), a “base correction” is needed to ensure that relationships in **G** and **A** are expressed relative to the same base or founder population, as explained in detail by Meuwissen et al. [38]. In the MEM (20) presented here, the variance component  for ***α*** and  for ***ε*** are kept separate. This strategy of keeping  and  separate can also be used in computing **H** by defining **G** as  instead of as .

Legarra et al. [11] and Christensen and Lund [14] gave alternative derivations of the matrix **H**. The derivation by Legarra et al. [11] uses the identity given by equation (7), which was also used here to develop the MEM for single-step Bayesian regression. Here, we reasoned that if **g** has a multivariate normal distribution, **g**_2_ and ***ε*** would be independent because they are also uncorrelated. Furthermore, we assumed that when conditioning on the observed marker information, the change in the distribution of **g**_1_ results directly from the change in the distribution in **g**_2_. However, one might argue that this assumption is not reasonable because when you condition on the observed value of **M**_2_, the change in the distribution of **g**_1_ results from the correlated change in the distribution of **M**_1_ and not from the change in the distribution of **g**_2_.

Christensen and Lund [14] did not rely on equation (7) to derive **H**, but they computed the mean of the missing genotypes conditional on the observed genotypes as:


However, this is valid only for multivariate normal variables, and multivariate normality is not a good approximation for the distribution of marker covariates. They also used an expression for the conditional variance of the missing genotypes that is valid only for multivariate normal variables. They did not explicitly recognize these approximations in their derivation, but pointed out that the conditional distribution of breeding values of the non-genotyped animals will only be approximately normal. Similar reasoning was also used by de los Campos et al. [39] who clearly showed that conditional on **M**_2_, **g**_1_ has a mixture of scaled-multivariate normal densities.

While we agree that conditional on **M**_2_, **g**_1_ has a mixture of scaled-multivariate normal densities, it must be noted that even the unconditional distribution of **g**_1_ is only approximately normal. Furthermore, in G-BLUP, what is being conditioned on is not the observed value of **M**_2_ but the observed value of **M**_2_**M**2′. To see this, note that there are many different **M**_2_ matrices that result in the same matrix for **G**, i.e. **M**_2_**M**2′, and thus the same G-BLUP breeding values. In other words, G-BLUP depends on **M**_2_ only through **M**_2_**M**2′, and therefore, all **M**_2_ that result in the same **M**_2_**M**2′ will also result in the same G-BLUP. However, **M**_2_**M**2′ is proportional to the covariance matrix of **g**_2_. Thus, it would be correct to say that in G-BLUP, what is being conditioned on is the covariance matrix of **g**_2_ changing from being proportional to **A**_22_ to being proportional to **M**_2_**M**2′. This conditioning may be thought of as a selection process, where the unselected samples of **g**_1_ and *g*_2_ have a multivariate normal distribution with null mean and covariance matrix , while in the selected samples, selection based on **g**_2_ results in its mean still being null but its covariance matrix changing to . Then, the correlated change in **g**_1_ is given by equation (10), which is a result from Pearson [40] that was also used by Henderson [16] to develop theory for BLUP under a selection model. However, given the selection process that we have described, the distribution of **g** may not be multivariate normal.

Hickey et al. [41] imputed genotypes for non-genotyped individuals so that all individuals have genotypes for fitting a MEM. In their case, imputation was undertaken using linkage and linkage disequilibrium information. However, they did not account for any imputation residual. Liu et al. [42] also have described a single-step MEM for combining information from genotyped and non-genotyped animals, which includes a residual polygenic component. Their analysis requires repeated multiplication of  by a vector, and an efficient algorithm has been developed for this multiplication. In another approach (Theo Meuwissen, personal communication, October 3, 2013), the LDMIP algorithm [43] was used to impute missing genotypes, and for each SNP where the genotype of an individual *i* was not known with certainty, a random effect with covariance matrix **G**_*LA*_ was fitted to account for the variability that is incompletely explained by the imputed genotype, where **G**_*LA*_ is the linkage analysis based covariance matrix [44]. One of the advantages of this approach is that imputation of genotypes by LDMIP at a locus *j* uses information from the genotypes of all linked loci, in addition to the genotypes at locus *j*. The implied imputation in SSBV-BLUP [11–14] and the method presented here, only uses genotypes at the current locus. Furthermore, best linear prediction is used for imputation, which is optimal only for normally distributed variables. In contrast, in LDMIP, conditional probabilities of the missing genotypes, given all observed genotypes at locus *j* and linked loci, that are computed approximately by iterative peeling and combine both linkage and LD information, are used to impute genotypes. Although the covariance matrix **G**_*LA*_ is easier to justify than the covariance of ***ε*** used here and in SSBV-BLUP, normality of the residuals of a single SNP covariate is more difficult to justify than normality of ***ε***. Use of mixture genetic models [45] addresses this weakness, but more work is needed to make these analyses efficient for routine use.

The single-step Bayesian regression approach presented here and SSBV-BLUP have the same appealing property that phenotype, genotype and pedigree information are combined in a single-step. Unlike SSBV-BLUP, SSBR is not limited to normally distributed marker effects; SSBR can be used with *t*-distributed marker effects, as in BayesA, and with mixture models, as in BayesB and BayesC *π*. Furthermore, it has the advantage that matrix inversion is not required. However, this comes at the expense of using MCMC methods that are computationally intensive, but these methods have the advantage that computing time and memory requirements increase linearly with the number of observations and number of markers. Thus, as demonstrated here, computing clusters can be used to parallelize and speedup MCMC analyses for routine applications.

## Electronic supplementary material

Additional file 1: **Efficient computation of**  **.** It is shown here how the matrix  of imputed marker covariates can be obtained efficiently by solving an easily formed sparse system of equations. (PDF 102 KB)
