# Supplementary material for: A class of Bayesian methods to combine large numbers of genotyped and non-genotyped animals for whole-genome analyses
Source: Genet Sel Evol. 2014 Sep 22;46(1):50. doi: 10.1186/1297-9686-46-50 (PMC4262255; doi:10.1186/1297-9686-46-50)
Supplement: Supplementary file 1 — Additional file 1: Efficient computation of . It is shown here how the matrix of imputed marker covariates can be obtained efficiently by solving an easily formed sparse system of equations. (PDF 102 KB) [file 12711_2013_2653_MOESM1_ESM.pdf]

Efficient computation of $\hat{\mathbf{M}}_1 = \mathbf{A}_{12}\mathbf{A}_{22}^{-1}\mathbf{M}_2$

Consider a random vector $\mathbf{u}' = [\mathbf{u}_1', \mathbf{u}_2']$ that has a multivariate normal distribution with null mean and covariance matrix $\mathbf{A}$. Then, the conditional mean of $\mathbf{u}_1$ given $\mathbf{u}_2$ is usually written as $\hat{\mathbf{u}}_1 = \mathbf{A}_{12}\mathbf{A}_{22}^{-1}\mathrm{u}_2$, where $\mathbf{A}_{ij}$ are the partitions of $\mathbf{A}$ corresponding to $\mathbf{u}_1$ and $\mathbf{u}_2$ [2]. We show here that $\hat{\mathbf{u}}_1$ can also be obtained as the solution to:

$$\mathbf{A}^{11}\hat{\mathbf{u}}_1 = -\mathbf{A}^{12}\mathbf{u}_2, \tag{1}$$

where $\mathbf{A}^{ij}$ is the sub-matrix corresponding to $\mathbf{A}_{ij}$ from $\mathbf{A}^{-1}$. Note that when $\mathbf{A}$ is the additive relationship matrix, $\mathbf{A}^{11}$ and $\mathbf{A}^{12}$ are very sparse and can be obtained efficiently [1]. Then, each column of $\hat{\mathbf{M}}_1$ can be obtained efficiently by taking $\mathbf{u}_2$ to be the corresponding column in $\mathbf{M}_2$ and solving equation (1) for $\hat{\mathbf{u}}_1$.

To show that $\hat{\mathbf{u}}_1$ can be obtained as the solution to equation (1), the multivariate normal density function for $\mathbf{u}$ is written as

$$\begin{aligned} f(\mathbf{u}) &\propto \exp[-\frac{1}{2}\mathbf{u}'\mathbf{A}^{-1}\mathbf{u}] \\ &\propto \exp[-\frac{1}{2}\{\mathbf{u}_1'\mathbf{A}^{11}\mathbf{u}_1 + 2\mathbf{u}_1'\mathbf{A}^{12}\mathbf{u}_2 + \mathbf{u}_2'\mathbf{A}^{22}\mathbf{u}_2\}]. \end{aligned}$$

Then, the density function for the conditional distribution of $\mathbf{u}_1$ given $\mathbf{u}_2$, which is also multivariate normal, is obtained by dropping terms that are free of $\mathbf{u}_1$ and completing the square:

$$f(\mathbf{u}_1|\mathbf{u}_2) \propto \exp[-\frac{1}{2}\{\mathbf{u}_1'\mathbf{A}^{11}\mathbf{u}_1 + 2\mathbf{u}_1'\mathbf{A}^{12}\mathbf{u}_2\}] \tag{2}$$

$$= \exp[-\frac{1}{2}\{(\mathbf{u}_1 - \hat{\mathbf{u}}_1)'\mathbf{A}^{11}(\mathbf{u}_1 - \hat{\mathbf{u}}_1) + \hat{\mathbf{u}}_1'\mathbf{A}^{11}\hat{\mathbf{u}}_1\}], \tag{3}$$

where for equation (2) to equal equation (3), $2\mathbf{u}_1'\mathbf{A}^{12}\mathbf{u}_2 = -2\mathbf{u}_1'\mathbf{A}^{11}\hat{\mathbf{u}}_1$ must hold for all values of $\mathbf{u}_1$, which implies that $\hat{\mathbf{u}}_1$ in equation (3) is the solution to equation (1). Note that the term $\hat{\mathbf{u}}_1'\mathbf{A}^{11}\hat{\mathbf{u}}_1$ is free of $\mathbf{u}_1$ and can be dropped from equation (3). Then, $f(\mathbf{u}_1|\mathbf{u}_2)$ becomes:

$$f(\mathbf{u}_1|\mathbf{u}_2) \propto \exp[-\frac{1}{2}(\mathbf{u}_1 - \hat{\mathbf{u}}_1)'\mathbf{A}^{11}(\mathbf{u}_1 - \hat{\mathbf{u}}_1)],$$

which is the density for a multivariate normal distribution with mean $\hat{\mathbf{u}}_1$, which can be obtained as the solution to equation (1).

An alternative derivation is obtained by substituting $\mathbf{A}_{12}\mathbf{A}_{22}^{-1}\mathbf{u}_2$ for $\hat{\mathbf{u}}_1$ in equation (1) giving

$$\mathbf{A}^{11}\mathbf{A}_{12}\mathbf{A}_{22}^{-1}\mathbf{u}_2 = -\mathbf{A}^{12}\mathbf{u}_2,$$

which must hold for all values of $\mathbf{u}_2$. This implies the identity:

$$\mathbf{A}^{11}\mathbf{A}_{12}\mathbf{A}_{22}^{-1} = -\mathbf{A}^{12}, \tag{4}$$

which can be obtained by writing $\mathbf{A}^{-1}\mathbf{A} = \mathbf{I}$ in partitioned form. In this product of partitioned matrices, the second column of the first row is:

$$\mathbf{A}^{11}\mathbf{A}_{12} + \mathbf{A}^{12}\mathbf{A}_{22} = \mathbf{0}.$$

Postmultiplying this expression by $\mathbf{A}_{22}^{-1}$ and rearranging gives equation (4). Thus, the use of equation (1) does not require multivariate normality.

References

[1] C. R. Henderson. A simple method for computing the inverse of a numerator relationship matrix used in prediction of breeding values. *Biometrics*, 32:69–83, 1976.

[2] D. A. Sorensen and D. Gianola. *Likelihood,Bayesian,and MCMC Methods in Quantitative Genetics*. Springer, 2002.
